# Threat-induced prosocial behavior: enhanced exogenous attention to protect others from harm

**DOI:** 10.1038/s41598-024-66787-3

**Published:** 2024-07-15

**Authors:** Maria Lojowska, Federica Lucchi, Manon Mulckhuyse

**Affiliations:** 1 Institute of Psychology, Leiden University, Leiden, The Netherlands; 2 Cognitive Psychology Unit, Institute of Psychology, Leiden University, Leiden, Netherlands

**Keywords:** Attention, Exogenous spatial cueing task, Threat, Pro-social behavior, Psychology, Human behaviour

## Abstract

As social animals, humans tend to voluntarily engage in pro-social behavior to prevent harm to others. However, to what extent prosocial behavior can be reflected at the level of less voluntary cognitive processes remains unclear. Here, we examined how threat to others modulates exogenous attention. Fifty-four participants performed an exogenous spatial cueing task where the participant’s performance determined whether electric shocks would be delivered either to themselves or to their anonymous co-participant. Threat of shock to the co-participant elicited orienting and reorienting responses that were faster than in the safe condition and did not differ from performance when participants avoided shocks to themselves. This attentional improvement was not due to speed-accuracy trade off and was associated with arousal, i.e., increased pupil dilation in both threat conditions. Together, these findings suggest that pro-social behavior triggers automatic attentional processes which may be relevant for providing immediate help without relying on reflexive processes.

## Introduction

Humans are unique in their capacity to help others. From participating in charity events and donations to rushing into burning buildings to save strangers, we help others at the cost to ourselves^1–3^. According to theories of directed altruism, distress to the other is thought to cause arousal in the observer who tries to alleviate this state through helping behavior^4^. Arousal has been associated with changes in cognitive functions such as enhanced attention to sudden and behaviorally relevant stimuli as an evolutionary adaptation to quickly detect and avoid harm when threat is directed to oneself^5–7^. Here, we examined to which degree attention performance in an exogenous spatial cueing task is affected when the safety of others is at stake.

As social animals living in groups, we are often faced with decisions to benefit the other at the cost to ourselves. Previous studies on economic decision making demonstrated that humans are willing to share their allocated resources with an anonymous other and to cooperate to benefit the whole group rather than keeping recourses for themselves^2,8^. Such pro-social behavior has particularly been observed when another person is exposed to potential harm^1,9^. For instance, when exposed to potential shocks, individuals are more likely to forego more of their earnings in order to avoid shocks to the other compared to themselves^10^. In yet another study, participants chose to allocate more pain to themselves than to the anonymous other supporting the view that humans are more sensitive to harm to the other than to oneself^11^. Recent studies have also suggested that individuals are more likely to risk a chance of receiving shocks to save the other when having limited time to decide^12^. Limited time to decide might reveal a predisposition of individuals to be more or less altruistic^13^. In the latter study, it was shown that an attentional bias towards an altruistic or selfish choice changed over time, reflecting a serial rather than a dual process. Note that most studies used a decision-making task to measure pro-social behavior. In the current study, we employed a task that did not require participants to make a trade-off between a pro-social and a selfish choice. Whether indeed pro-social behavior is driven by a reflexive (vs. reflective) response is till up for debate^8,14^.

One way to measure reflexive or less controlled cognitive behavior involves assessing exogenous attention. Exogenous attention is considered to be a reflexive cognitive process and refers to orienting to salient, but task irrelevant stimuli^15^. In exogenous attention, a salient stimulus, such as a sudden flashing light, captures attention involuntarily^15^. Reorienting, on the other hand, occurs when attention shifts voluntarily from an attended location to an unattended location^16^. Previous research has shown that both processes are modulated when individuals are exposed to aversive conditions. That is, orienting and reorienting improved when individuals were exposed to monetary punishment if their attentional performance fell below a set threshold^17^. Improvement in attentional processes under threat has been suggested to aid optimal detection of relevant information in the environment to inform optimal responses^6,18^. However, if and to what extent exogenous attention improves to prevent harm to the other, remains unclear.

In addition to attentional improvement, individual exposure to threat has been characterized by the mobilization of autonomic physiological responses^6,19^. During anticipatory threat, sympathetic nervous system becomes activated to prepare for potential action, which is reflected in an increase in skin conductance and pupil dilation^20^. Autonomic mobilization and attentional changes have been suggested to be co-regulated by a common neural mechanism involving the amygdala^21^. Autonomic responses have also been recruited not only during threat to oneself, but also when exposed to a threat to another person^12,22,23^. Exposure of others to shocks has been found to recruit autonomic physiological responses (i.e., increase in skin conductance) and brain networks that are also activated during individual exposure to threat^12^. The observer’s automatic arousal is thought to drive helping actions to alleviate distress in both the other person and themselves^4,24^. Indeed, higher arousal indexed by elevated skin conductance predicted voluntary (deliberate) helping behavior in the observers who chose to endure pain from the other^22,23^. Following these findings, we were specifically interested whether we would find enhanced sympathetic arousal under threat, irrespective of whose safety was at stake.

The goal of the current study was to investigate whether exogenous attention is affected when punishment to the other can be avoided. To test this prediction, participants performed an exogenous spatial cueing task in dyads under threat^25^. Depending on their performance, participants could avoid threat, i.e., unpleasant electric shocks, to oneself or to the anonymous co-participant. We used pupil dilation as a measure of sympathetic autonomic activation^20^ and expected to find increased pupil dilation when threat was directed to the participants themselves or to the other compared to the safe condition. Building upon previous attention research, our expectation was to replicate the typically observed pattern of better performance (i.e., faster reaction times and higher accuracy) in valid trials, in which the target is presented at the cued location compared to invalid trials, in which the target is presented at the opposite, un-cued location^26^, indicating attentional capture by the cue. We had subsequently two main hypotheses. First—and similar to Engelmann and Pessoa^17^—we predicted that if threat to oneself enhances orienting and reorienting, we should observe respectively faster reaction times and higher accuracy for valid and invalid trials compared to the safe condition. Second, if exogenous attention improves to protect the other from harm, similar results should be expected when threat was directed to the other.

## Materials and methods

### Participants and ethics

Power calculation for the behavioral effects in mixed models was performed using power simulation in R using the simr package^27^. Following recent recommendations for power size calculations (based on available resources^28^), the maximal sample size feasible to test was set to 55 participants. The simulation revealed that the minimum effect size (representing the difference in mean RT between Shocks to Self or Shocks to Other conditions and to No shocks condition) that can be detected with this sample size and at power of 80% was *B* = 1.6. In total, we tested 58 dyads in the current experiment to account for unexpected loss of data. Four dyads were excluded from the analysis due to technical issues, resulting in the final sample of 54 participants. Mean age of the participants that performed the task (Performers only, see below) was 20.2 years (SD: 2.38), and there were 46 female participants and 8 male participants.

Participant inclusion criteria were: age between 18 and 35 years old, normal or corrected-to-normal vision, no colorblindness, no history of psychiatric or neurological conditions, sufficient understanding of English, no medical devices such as heart pacemaker. The study was approved by the ethics committee of the Faculty of Social Science of Leiden University, The Netherlands (approval number: V2-3393). The experimental procedures were performed in accordance with the Helsinki declaration. All participants provided written informed consent and received financial compensation or course credits for their participation (8.50 euro or 2 course credits). In addition, participants earned extra money with their decisions in the Social Value Orientation Task (range from 0.63 to 1.02 euro).

### Threat induction and physiological measures

Threat was induced through a chance of receiving unpleasant, but not painful electric shocks. Electric shocks were delivered transcutaneously through the participant’s third and fourth distal phalanges of the non-dominant hand using Digitimer Constant Current Stimulator DS7 (www.digitimer.com) and standard Ag/AgCl electrodes. The duration of the electric stimulation was 200 ms, with a 50 Hz repetition of 250 µs pulses. The intensity of electric shocks varied between 0.6 and 10 mA. Shock intensity was adjusted at the individual level to ensure the shocks were unpleasant, but not painful. Shock calibration was performed using a standard shock calibration procedure comprising the delivery of several sample shocks, starting with 1.2 mA. Following each sample shock administration, the participants verbally rated how unpleasant the shock was for them, on a scale from 1 (not unpleasant at all) to 5 (very unpleasant), after which the shock intensity was adjusted with increments of 0.6 mA.

When participants rated the shock intensity as 5, they were subsequently asked whether the shock was painful. If the answer was yes, the shock intensity was downregulated by one increment (i.e., 0.6 mA). The procedure stopped when a sample shock was rated as very unpleasant, but not painful, or if the maximum shock intensity was reached (i.e., 10 mA). Furthermore, participants were asked whether they would be fine with receiving this shock intensity during the experiment. Unpleasant but not painful shocks levels were commonly found with 4 exposures to sample shocks. The final shock intensity obtained with this method was used in the main task.

To assess whether our threat manipulation was successful, we recorded pupil dilation of the participant that performed the task (the Performer). Pupil dilation was used for offline assessment of sympathetic activity indexing arousal. Pupil dilation was acquired using Tobii eye-tracker (version: X3-120), with a sample rate of 120 Hz.

### Experimental procedure

Two participants took part in the current experiment. The Performer actively performed the experiment, whereas the Receiver was a passive recipient of potential shocks depending on the Performer’s performance. All participants received the information brochure with the study description and exclusion criteria at least 24 h before the experiment. No deception was used in the study. Participants were informed that they could be assigned an active or passive role in the experiment. They were also informed that if they had been assigned an active role of the Performer, their performance in the task would have real consequences (in terms of shocks) to them or to the Receiver. Participants in each dyad were randomly assigned to the role of the Performer or to the role of the Receiver.

**Figure 1:**
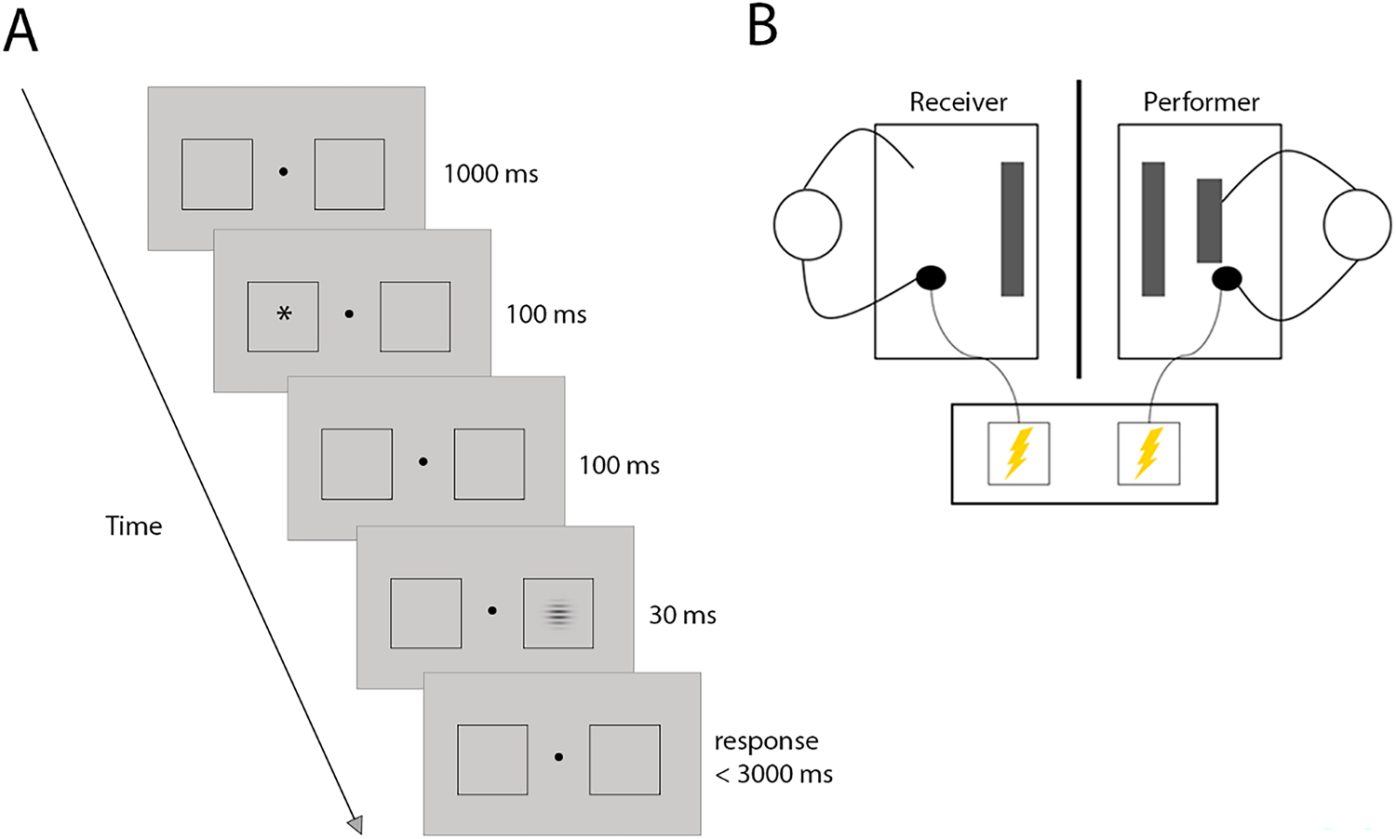
(**A**) Exogenous attention cueing task—example of an invalid trial. Performers had to indicate as quickly and accurately as possible whether the orientation of the target, a Gabor grating, was horizontal or vertical. Preceding the target, a cue—an asterisk—was presented either on the same side of the target (valid trials) or the opposite side of the target (invalid trials). (**B**) Experimental set-up. Participants remained mutually anonymous through an opaque divider separating them from each other.

Participation was anonymous, i.e., although the participants were in the same room, they were unable to see each other during the experiment (Fig. 1B) and they were informed that their identity would not be revealed to the other participant to avoid reputation concerns. Furthermore, during the experiment, participants were asked to wear ear mufflers so that they were unable to hear each other. To ensure that the Performer believed that the other participant was actually present and subjected to the experimental treatments, they were clearly informed that no deception would be used, and the shock calibration procedure of the Receiver took place in the same room behind the partition while the Performer was waiting (wearing ear muffler). To further preserve anonymity, the two participants were welcomed at two different locations at the university and entered the lab consecutively at different times. Before entering the lab, both participants read the information brochure with information about the assigned role in the task, were given time to answer questions, and signed the informed consent. The Performer was informed that they would carry out the experiment, that depending on their performance either them or the Receiver could receive an electric shock. The Receiver was informed that their role in the task was passive, and that depending on the performance of the Performer they could receive shocks during the experiment.

The Performer was welcomed first in the lab. First, shock electrodes were attached to their fingers and the Performer underwent a shock calibration procedure (as outlined above). The Performer was then asked to wear mufflers and the Receiver was asked to enter the lab. Subsequently, shock calibration procedure was performed for the Receiver. Upon its completion, the Receiver was asked to follow along the task on the screen in front of them. The Performer’s chin was located on the chin rest to ensure good performance of the eye tracker. The experiment began with the calibration of the eye tracker after which the Performer followed the instructions on the screen.

The Performer performed an exogenous attention cueing task (see below). The experiment started with the practice session, followed by the training session and the main task. The practice session consisted of four blocks of 32 trials, so that the learning effects were less present in the subsequent main task. After each block, both participants were informed via written information on the screen about the Performer’s performance: their mean reaction time (RT) and accuracy. In calculation of the mean RT, only correct trials were included, i.e., trials in which a correct response was given within a max. 3 s period following the presentation of the grating. The practice session was followed with a training session consisting of 32 trials. The mean RT calculated from these trials was used in the calculation of the threshold RT used in the subsequent main task. No shocks were delivered during the practice and training sessions.

Subsequently, the Performer performed the main task. The main task consisted of the following conditions: Shocks to Self, Shocks to Other, No shocks (safe condition) organized in blocks of trials. At the beginning of each block, participants were informed that either the Performer (Shocks to Self), the Receiver (Shocks to Other), or no-one (No shocks) would receive the shocks at the end of it. The Performer was instructed that to avoid the shock at the end of the block, their responses in that block must be at least 75% correct, and faster than their individual RT threshold (calculated in the training session). The RT threshold was calculated by adding an addition 50 ms to the mean RT from the training session. At the end of each block, the participants were informed about the Performer’s performance (mean RT and accuracy) and whether the shock would be delivered or not. In case of a shock, participants were asked to prepare for the shock, and both were notified when the shock was delivered. Subsequently, the Performer was asked to rate how afraid they were for themselves (in the shocks to self condition), or for the receiver (in the shocks to other condition) to receive the shocks during that block, on a scale from 1 (not afraid at all) to 5 (very afraid). to test for shock habituation during the task, in the shock to self condition, the performers were additionally asked to rate the unpleasantness of the received shock if they had received one, on a scale from 1 (not unpleasant at all) to 5 (very unpleasant). In total, only 5 participants (3 Performers and 2 Receivers) received shocks due to the Performer not reaching the performance threshold (Fig. S1). After each block of trials, there was an 8 s break during which participants were presented with a screen with a fixation dot in the middle of it. Each condition was organized into 4 blocks of 16 trials (64 trials per condition), resulting in a total of 12 blocks. The order of the conditions were counterbalanced between the participants.

At the end of the experiment, the Receiver was first accompanied outside the laboratory and debriefed. In the meantime, the Performer was asked to fill in the following questionnaires: Social Value Orientation task (SVO, ^29^), the Trait Anxiety Inventory^30^, the Toronto Empathy Questionnaire^31^, and Behavioural Inhibition System scale^32^. They were also asked to complete a modified version of the SVO questionnaire in which they were presented with a hypothetical scenario of how likely it would be for them or the other hypothetical person receive the shocks, and how much they would want to reduce the chance of receiving the shocks for themselves and for the other person. They were also asked to complete an exit questionnaire and debriefed about the purpose of the experiment.

### Exogenous attention cueing task

Each trial started with the presentation of the black fixation dot at the centre of the screen, on the grey background and with two white placeholders presented to the left and right of it (Fig. 1A). Subsequently, a cue consisting of a white asterisk was presented for 100 ms in one of the placeholders. Following an inter-stimulus interval of 100 ms, the target, i.e., a Gabor grating was presented within one of the placeholders for 40 ms. Half of the trials in the task contained the gratings oriented horizontally and the other half contained the grating oriented vertically. The Performer was asked to indicate as fast and accurate as possible whether the grating was vertical by pressing the ‘m’ button with their right middle finger, or horizontal, by pressing the ‘n’ button with their right index finger on the keyboard. The next trial started after the response was given or after 3 s had passed. In the practice session, when the response was incorrect or too late, the fixation dot turned red. No feedback was given in the training session and in the main task. The task was programmed in Open Sesame [version: 3.3.14; ^33^]), displayed on the screen with a 1024 × 768 resolution, presented and at the distance of 57 cm from the Performer’s eyes.

### Physiological and behavioral analyses

The analysis of pupil dilation was performed offline using in-house software implementation in MATLAB (Matlab, R2018b, The MathWorks, Inc), allowing for visual assessment and removal of signal artifacts. Blinks in pupil data were removed from the signal using linear interpolation. Pupil dilation was calculated as an average of pupil dilation for the left and right eye, unless data for one of the eyes were missing in case of which only pupil dilation from one eye was included. Due to missing or very noisy data, a total of 48 participants (out of 54 participants that were included in the behavioral analyses) were included in the analysis of pupil responses. Within this sample, noisy parts of the signal—if present—were excluded from the analysis. To assess the change in arousal due to threat induction procedure, pupil responses were calculated for each block of trials.

Statistical analyses were carried out in R (Version 3.5.1; R Core team, 2016). The analyses were performed using linear mixed-effects models as implemented by the lmer function (lmer4 package, version: 3.3.1^34^). Mixed models were used to account for repeated measures across individuals. The model for pupil dilation included fixed effects for threat condition (Shocks to Self, Shocks to Other, No shocks), block number within each condition (1–4), order of the conditions (1–3) as a covariate, and pupil dilation as a dependent variable. Following our preregistration (https://osf.io/n9hrk), two separate types of models were used to test behavioral results. The first types of models tested the effects of threat conditions separately on performance (RT, accuracy) in valid and invalid trials (indexing orienting and reorienting, respectively). These models included fixed effects for the following predictors: threat condition, block number, trial validity (valid, invalid), order of the conditions as a covariate, and RT and accuracy as dependent variables (in two separate models). In the calculation of RT, only correct trials were included. Trials in which responses were shorter than 100 ms or trials on which responses were longer than the 3000 ms response window, were excluded from the analyses. In the models assessing accuracy, all trials were included. Trials with responses shorter than 100 ms or longer than the response window (> 3000 ms) were coded as incorrect. The second type of behavioral models we used tested for the cue validity effect, i.e., the difference in performance between invalid and valid trials. This type of models included threat condition, block number and order of conditions as fixed effects, and the difference in RT (and accuracy) between valid and invalid trials as dependent variable. Fixed effects for the interactions between threat condition, block number (and cue validity in the first type of models) were also included in the models. Between-subject variance was modelled by including a per-participant random adjustments to the fixed intercept (‘random intercept’). Continuous predictors were centered and all categorical predictors were coded using sum coding. Accuracy was a binary measure for which a logistic mixed-model, as implemented in the glmer function (lmer package, version: 3.3.1^34^), was fitted. As a general strategy, we first used an omnibus model investigating the main and the interaction effects where *p*-values were determined using Type 3 Likelihood Ratio Tests implemented in the *mixed* function of the package *afex* ^35^. In linear mixed effects models, point estimates (B) were used as a measure of the magnitude of the effects, and their corresponding *p* values were obtained using *lmerTest* package^36^. In logistic mixed-effect models, odds ratio and their 95% CI were calculated as a measure of significance of the effects. For the close-to-significant results we calculated Bayes Factors (BF_10_) to measure the extent to which the data provide evidence in favor of the absence of an effect using *BayesFactor* package in R^37^.

## Results

### Physiological and subjective responses to shocks

**Figure 2:**
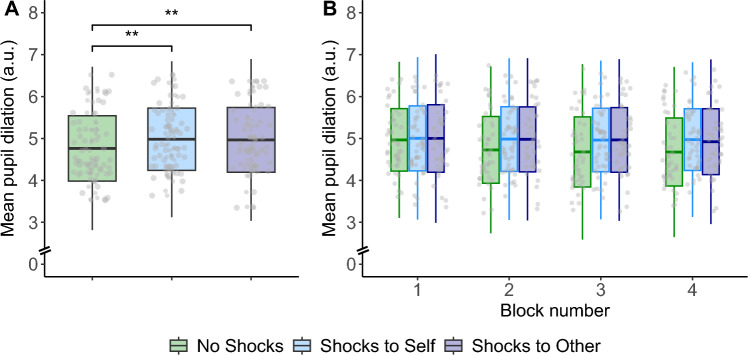
(**A**) Larger pupil dilation when participants themselves (Shocks to Self) and when the co-participants (Shocks to Other) were exposed to potentials shocks compared to the safe (No Shocks) condition. (**B**) Pupil dilation remained high and stable across time (blocks) during both threat conditions (Shocks to Self, Shocks to Other), but decreased over time in the safe condition. a.u., arbitrary units. Box plots represent mean and ± SD of the mean. The whiskers represent ± 2.5 SD of the mean.

To determine the effectiveness of our threat manipulation, we first examined pupil dilation in our task. This was done to test whether sympathetic responses—indexed by pupil dilation and commonly observed in threat paradigms^20^—were observed when the participants (Performer or Receiver) were exposed to potential shocks. We found that compared to the safe (No Shocks) condition, exposure to potential shocks evoked larger and stable pupil dilation. This was observed in conditions when the participants themselves (Performer) as well as when the co-participants (Receiver) could receive potential shocks. Specifically, there was a main effect of threat condition (Shocks to Self, Shocks to Other, No Shocks), *χ*^2^(1) = 34.30, *p* < 0.001 (Fig. 2A). Pupil dilation was larger when participants themselves were exposed to potential shocks compared to the safe condition, *B* = − 0.11, SE = 0.01, *χ*^2^(1) = 32.23, *p* < 0.001. Importantly, increased pupil dilation was also observed when the co-participant could receive shocks relative to the safe condition, *B* = − 0.10, SE = 0.01, *χ*^2^(1) = 79.65, *p* < 0.001, but there were no significant difference in pupil dilation when the participants themselves and the co-participants were under potential shocks, *B* = − 0.006, SE = 0.009, *χ*^2^(1) = 0.39, *p* = 0.53.

In addition to the overall larger pupil dilation in both threat conditions, these responses remained high and stable across time (block 1–4), *χ*^2^(2) = 27.91, *p* < 0.001. Specifically, when the participants were exposed to potential shocks themselves, pupil dilation remained higher and stable across the time compared to the safe condition, *B* = 0.04, SE = 0.009, *χ*^2^(1) = 16.57, *p* < 0.001 (Fig. 2B). Similar pattern was observed when the co-participant was exposed to potential shocks where pupil dilation also remained high and stable across time compared to the safe condition, *B* = − 0.03, SE = 0.009, *χ*^2^(1) = 11.81, *p* < 0.001. Crucially, whereas no differences in pupil dilation across time was observed when the participants themselves and when the co-participants were exposed to potential shocks, *B* = − 0.007, SE = 0.008, *χ*^2^(1) = 0.71, *p* = 0.40, pupil dilation in the safe condition showed a decreasing trend over time, *B* = − 0.09, SE = 0.01, *χ*^2^(1) = 25.74, *p* < 0.001. Full model results are provided in Table S1.

In addition to physiological responses, we also analyzed the subjective ratings of fear that participants experienced for themselves and for the co-participant during the task. There was no difference in fear ratings when the participants themselves and the co-participant were exposed to potential shocks, *B* = − 0.15, SE = 0.10, *χ*^2^(1) = 2.38, *p* = 0.12. However, and in contrast to the physiological results, the ratings decreased over time irrespective of threat condition, *B* = − 0.25, SE = 0.04, *χ*^2^(1) = 42.90, *p* < 0.001, from an average rating of M = 3.42 (SD = 0.80) in block 1, to an average rating of M = 2.68 (SD = 1.00) in block 4 (Fig. S2).

### Behavioral responses

**Figure 3:**
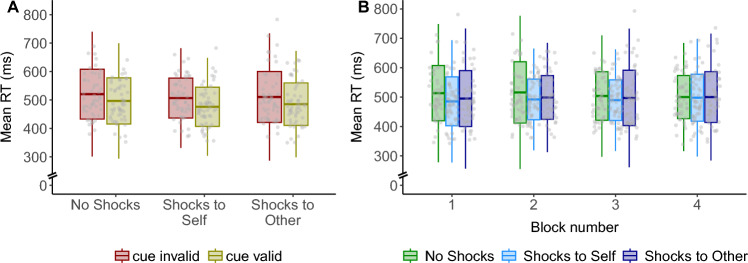
Reaction times for valid and invalid trials in the exogenous attention cuing task. (**A**) Faster responses when participants themselves were exposed to potential shocks (Shocks to Self) and when the co-participant was exposed to potential shocks (Shocks to Other) compared to the safe condition (No Shocks). (**B**) Faster and stable across time responses were observed for the both threat conditions. RT, reaction time; ms, milliseconds. Box plots represent mean and ± SD of the mean. The whiskers represent ± 2.5 SD of the mean.

Having observed increased pupil responses when shocks were directed to the co-participant as well as to participants themselves, we subsequently examined how exogenous attention is affected in these two conditions. First, and following previous research^26^, we found a significant main effect of cue validity on RT. Faster responses were observed in valid (M = 486.16 ms) compared to invalid trials (M = 512.36 ms), *B* = 13.18, SE = 1.34, *χ*^2^(1) = 95.82, *p* < 0.001, confirming that our cue manipulation was successful. Subsequently, we also found a significant main effect of threat condition on RT, *χ*^2^(2) = 26.93, *p* < 0.001. Follow-up analyses showed that responses were faster when participants themselves were exposed to shocks compared to the safe condition, *B* = 8.26, SE = 1.65, *χ*^2^(1) = 25.11, *p* < 0.001 (Fig. 3A). Importantly, when the co-participant was exposed to potential shocks, responses were also faster than in the safe condition, *B* = 5.90, SE = 1.75, *χ*^2^(1) = 11.38, *p* < 0.001. There was no significant difference in RT when participants themselves and when the co-participants were exposed to potential shocks, *B* = -2.94, SE = 1.54, *χ*^2^(1) = 3.65, *p* = 0.06 (BF_10_ = 0.15, suggesting a moderate evidence in favor of the absence of the effect). The interaction between threat condition and cue validity was non-significant, *χ*^2^(2) = 4.42, *p* = 0.11.

To further understand whether the effects of threat were present for both orienting and reorienting responses, and in line with our preregistration plans (https://osf.io/n9hrk), we analyzed the effect of threat on RTs separately for valid and invalid trials. The simple effect analysis revealed that a significant main effect of threat on RT was observed in valid, *χ*^2^(2) = 20.72, *p* < 0.001, and invalid trials, *χ*^2^(2) = 8.63, *p* = 0.01 (full model results in Supplementary Material Table S2).

For valid trials, responses were faster when participants themselves were exposed to shocks compared to the safe condition, *B* = 10.01, SE = 2.17, *χ*^2^(1) = 21.19, *p* < 0.001, and when the co-participants was exposed to potential shocks compared to the safe condition, *B* = 6.03, SE = 2.38, *χ*^2^(1) = 6.42, *p* = 0.01. Responses on valid trials were also faster when threat was directed to one-self compared to the other, *B* = − 4.32, SE = 2.04, *χ*^2^(1) = 4.48, *p* = 0.03.

For invalid trials, responses were also faster when participants were exposed to potential shocks themselves (M = 506 ms) compared to the safe condition (M = 520.47 ms), *B* = 6.61, SE = 2.48, *χ*^2^(1) = 7.09, *p* = 0.007, and when the co-participant was exposed to potential shocks (M = 510.51 ms) compared to the safe condition, *B* = 5.78, SE = 2.56, *χ*^2^(1) = 5.10, *p* = 0.02. The difference in responses on invalid trials when threat was directed to oneself vs. the other was non-significant, *B* = − 1.62, SE = 2.30, *χ*^2^(1) = 0.50, *p* = 0.48 (see Table S3 for the average mean responses and their standard deviations).

In addition to the overall faster responses in both threat conditions, we also found that these responses remained stable across time (block 1–4). Specifically, there was a significant interaction between threat condition and time, χ^2^(2) = 7.82, p = 0.02 (Fig. 3B). Responses were faster and remained stable across time when participants were themselves exposed to potential shocks compared to the safe condition, B = − 3.89, SE = 1.47, χ^2^(1) = 7.02, p = 0.008. A similar pattern emerged when the co-participant was exposed to potential shocks, i.e., responses during this condition were faster and remained stable across time compared to the safe condition, B = -3.24, SE = 1.56, χ^2^(1) = 4.32, p = 0.037. RT during the safe condition showed a decreasing trend over time, B = − 4.75, SE = 2.30, χ^2^(1) = 4.28, p = 0.04. The interaction between threat condition and time did not differ significantly between valid and invalid trials, χ^2^(2) = 4.42, p = 0.11. Full model results are provided in Supplementary Material Table S2).

**Figure 4:**
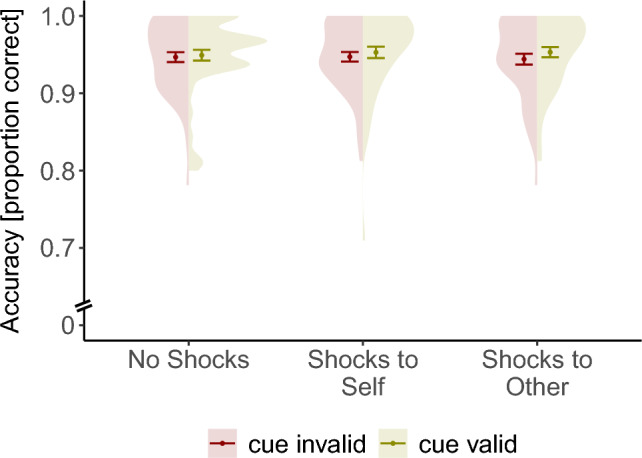
No difference in accuracy was observed between the three conditions, also not as a function of cue validity. Violin shapes represent the distribution of individual data points. Error bars represent standard errors of the mean.

To complement our analysis of RT, we subsequently analyzed accuracy (Fig. 4). There was no significant difference in accuracy between valid and invalid trials *χ*^2^(1) = 2.40, *p* = 0.12. The main effect of threat condition on accuracy was also not significant, *χ*^2^(2) = 0.76, *p* = 0.68, also not as a function of time, *χ*^2^(2) = 2.31, *p* = 0.31, indicating that the faster reaction times in the threat conditions vs. safe conditions were not due to a speed-accuracy trade-off^38^. The interaction between threat condition and cue validity was also not significant, *χ*^2^(2) = 0.44, *p* = 0.80. There was a significant interaction between cue validity (valid vs. invalid trials) and time, OR = 0.9, 95% CI [0.82, 0.97], *χ*^2^(2) = 7.05, *p* = 0.0.008, where the odds of responding correctly increased with time for valid trials, OR = 1.18, 95% CI [1.04, 1.34], χ^2^(1) = 8.31, *p* = 0.004, but not for invalid trials, OR = 0.95, 95% CI [0.85, 1.07], χ^2^(1) = 0.68, *p* = 0.41 (full model results are given in Supplementary Material Table S2). These results support the conclusion that stimulus-driven spatial attention improves to help the anonymous other avoid threat. Threat facilitates both orienting and reorienting responses and in a manner comparable to individual exposure to threat.

Following our preregistration plans, we also tested the cue validity effect, i.e. the difference in RT between invalid and valid trials between the threat conditions. The analysis of the difference scores yielded a non-significant difference between the conditions, also not as a function of time (*ps* > 0.05, Table S4). These results show that threat did not specifically affect orienting or reorienting, but that it resulted in an overall improvement relative to the safe condition.

## Discussion

When confronted with a deliberate choice, humans often choose prosocial actions aimed at preventing harm to others^1–3^. The current study demonstrates that threat to others also enhances more reflexive processes such as exogenous attention to avert others' exposure to pain. In the exogenous cueing task, threat of shocks to other improved both orienting and reorienting responses compared to the safe condition. This behavioral improvement was accompanied by heightened arousal evidenced by an increase in pupil dilation. These results suggest that prosocial behavior can be observed at the level of more reflexive cognitive responses, and that the integration of cognitive and autonomic processes observed during individual exposure to threat may be also observed to prevent harm to the other.

We found that both orienting and reorienting improved under threat. Specifically, faster responses in valid and invalid trials we observed to avoid shocks to participants and to others, compared to the safe condition. Orienting in an exogenous spatial cueing task is thought to represent attentional capture, which is considered to be an involuntarily driven process^26,39^. Anticipatory threat state is characterized by hypervigilance, and enhanced orienting during this state has been proposed to aid optimal detection and fast responses to potentially relevant stimuli^40,41^. Faster responses in valid trials when participants faced threat compared to when the others faced threat may suggest a stronger impact of self-directed threat on orienting. Reorienting is a more voluntarily driven process, although detecting behaviorally relevant stimuli outside the focus of attention is considered a form of bottom-up driven processes^16^. Detecting the target in our paradigm was behaviorally relevant since slower detection meant punishment, either to the participant themselves or to the others. Faster responses in the invalid trials in both threat conditions could, therefore, represent faster detection of the targets^16^ or faster voluntarily reorienting of attention to the targets ^17^. Together, our findings suggest that an exposure to threat facilitates automatic orienting towards salient information, as well as more voluntarily re-orienting towards behaviorally relevant information to avoid harm for the self or others.

Improvement in attentional processes has so far been demonstrated as an adaptive mechanism to avoid threat to oneself^6,40,42,43^. Specifically, individuals exposed to potential financial penalties showed improvement in orienting and reorienting responses that allowed them to evade these consequences^17^. Our study compliments and extend these findings in two distinct ways. First, we demonstrated attentional improvement to physical threat, supporting the view that exogenous attention improves when the wellbeing of an individual is compromised, irrespective of threat modality. Second—and crucially—we observed that exogenous attention improves to prevent harm to the anonymous co-participant. In the literature, prosocial behavior has mainly been supported by studies in which participants made deliberate decisions to help others under threat. For example, participants were willing to forego more of their own earnings in order to reduce harm of shocks to the other compared to themselves^10^. Here, we show that prosocial behavior can be manifested at the level of less voluntary, attentional processes.

Threat resulted in improved performance on reaction time, but had no effect on accuracy. This is inconsistent with Engelmann and Pessoa ^17^ who found an effect on accuracy performance. A possible explanation for this difference could be that the cue in their study - although a peripheral onset, was predictive of the target location (70% valid). Hence, this induced voluntarily driven shifts of attention. It is known that voluntary attention affects reaction time as well as accuracy in spatial cueing, whereas involuntary attention mainly affects reaction time^44^. In our study, we secured measuring involuntary attention by using an uninformative peripheral onset cue that consequently affected reaction time and not accuracy. In addition, participants were presented with an individual reaction time threshold, but not with an individual accuracy threshold. The accuracy threshold was set at 75% correct for all participants and the results showed a ceiling effect, indicating that task difficulty was low. Since we wanted to establish an accurate reaction time threshold in the training session, participants performed 4 blocks of practice to avoid learning effects reflected on reaction time. However, this might have also led to the relatively high accuracy performance. Knowing that their accuracy was well above 75%, the participants might have prioritized being fast over accurate, although accuracy remained high and stable during the blocks. More importantly, this latter result indicates that the faster reaction times were not due to speed-accuracy trade-off^38^.

The high performance in the Shock to Other condition might also indicate that the Performer felt a strong sense of responsibility and sense of agency, the feeling of being in control of one’s own actions ^45,46^. It is known that sense of agency is higher when one has the feeling of being in control of the situation^47^. Feedback about their good performance after each block, might have enhanced this feeling. In addition, in our set-up there was no beneficial outcome for the Performer when the co-participant was shocked. Possibly, this might have increased the sense of responsibility of the Performer, which is reflected in the very low number (in total 2 out of 42 participants) of shocks that the Receiver received due to bad performance of the Performer. Sense of agency and sense of responsibility might therefore affect automatic cognitive processes, such as exogenous attention.

The improvement in exogenous attention during an exposure to threat of the-coparticipant was associated with increased pupil dilation. Interestingly, the level of attentional improvement and pupil dilation did no differ when threat was directed to the other compared to the self. Individual threat exposure co-regulates attention and autonomic responses, fostering sensory intake and readiness for potential action based on environmental cues^19,40,48 ^. Previous studies have shown that autonomic responses, i.e., changes in heart rate, were also observed when individuals were faced with a choice to help the other avoid shocks^12^. According to theories of directed altruism, distress in another triggers autonomic stress responses in the observer through an automatic transfer of emotions^24^. Although emotional transfer is strongly triggered in direct interactions (e.g., through facial expressions), it has also been observed in indirect human interactions when participants do not see each other^49^. It is therefore plausible that pupil dilation in our study represent distress to the state of the co-participant. Overall, our results support the view that similar autonomic and cognitive processes may be in place to protect self and the other from immediate harm. Future studies should include direct participant interactions (while account for reputation effects) and measure distress in both parties to further understand the role of emotional contagion in mobilizing autonomic and cognitive processes to aid others. Moreover, sense of agency and sense of responsibility might change in the clear presence of another human being.

Some interpretational issues should be considered when evaluating the current findings. One could argue that increased responses in our task represent an improvement in motor responses rather than attentional effects. Indeed, anticipatory threat has been conceptualized as a state of motor preparation characterized by faster responses to sudden visual input if they warrant threat avoidance^50,51^. Although we cannot exclude the contribution of motor preparation in our task, the specificity of our results to attentional effects can be supported by two pieces of evidence. First, an individual exposure to punishment has previously increased accuracy of orienting and reorienting responses, which in addition to our threat-related improvement in reaction times supports the specifically of our findings for attentional processes^17^. Second, a general facilitation of motor responses would likely lead to faster responses in threat conditions (to self and to the other) at the cost of accuracy. If this was the case, we should observe a speed-accuracy tradeoff with faster RTs in the threat conditions, but more errors compared to the safe condition. This is not supported by our results as the three conditions do not differ in their accuracy.

As social animals, we often make deliberate choices to help others in need. Here, we demonstrated that pro-social behaviour can also be reflected at the level of more involuntary cognitive processes. Exogenous attention improved to help an anonymous other avoid harm, which did not differ when participants avoided harm to themselves and was associated with concomitant arousal. These findings potentially indicate that similar autonomic and cognitive mechanisms might have evolved for self-protection and to assure the welfare of others. The automatic nature of these responses may be crucial in providing immediate help without relying on available mental resources or reflective processes.

### Supplementary Information

Supplementary Information.

## Data Availability

The data for the current study are publicly accessible at https://osf.io/v8sf4/. The code for the current study is publicly accessible at https://osf.io/v8sf4/. The experimental task is publicly accessible at https://osf.io/v8sf4/. There is a preregistration for the current study here: https://osf.io/n9hrk.
