# Supplementary material for: Threat-induced prosocial behavior: enhanced exogenous attention to protect others from harm
Source: Sci Rep. 2024 Jul 15;14:16252. doi: 10.1038/s41598-024-66787-3 (PMC11251053; doi:10.1038/s41598-024-66787-3)
Supplement: Supplementary file 1 — Supplementary Information. [file 41598_2024_66787_MOESM1_ESM.docx]

**Supplementary Material**

**Threat-induced prosocial behavior:**

**Enhanced exogenous attention to protect others from harm.**

Maria Lojowska, Federica Lucchi, Manon Mulckhuyse

Institute of Psychology, Leiden University, Leiden, The Netherlands


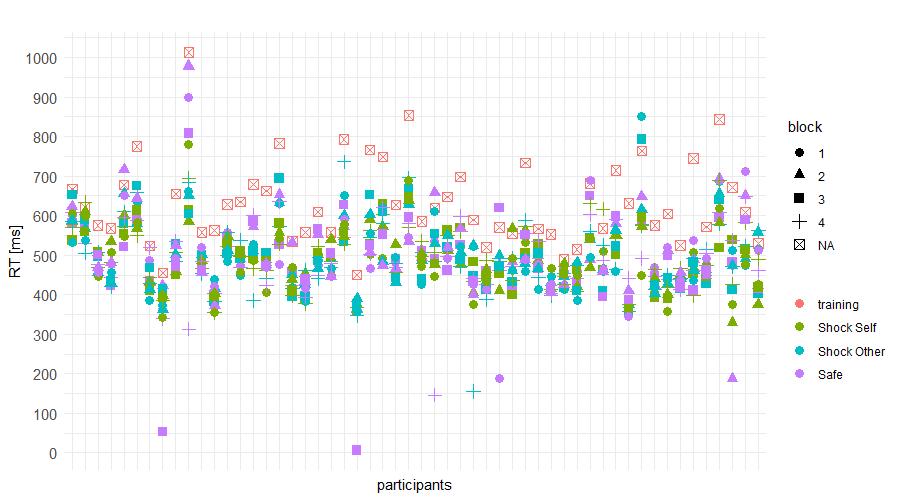


**Figure S1.** Mean reaction times during each condition (different colors) and blocks (different shapes). RT for each participant from the training phase is displayed with additional 50 ms (threshold RT, red square with a cross in it). In the actual attention cuing task, participants were instructed to perform faster than their threshold RT, and be correct on 12 out of 16 trials in each block. If this requirement was not met, participants themselves (in the Shocks to Self condition) or the co-participant (in the Shocks to Other condition) received electric shocks at the end of a given block of trials. No shocks were delivered in the Safe condition. Only 5 participants performed (either on Shocks to Self or Shock to Other conditions) above their threshold RT: 3 participants received shocks themselves, and 2 co-participants received shocks (1 co-participant in one block of trials, and 1 co-participant in two different blocks of trials).


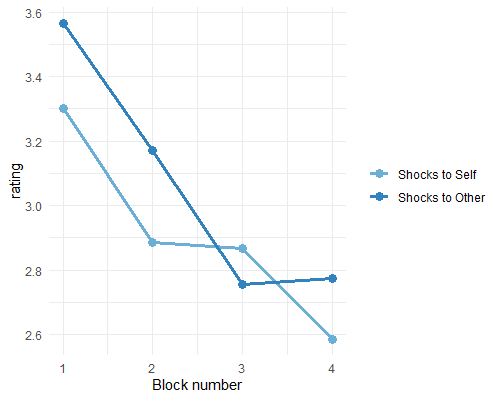


**Figure S2.** There was no difference in the ratings of fear when participants themselves (Shocks to Self) and when the co-participant (Shocks to Other) were exposed to potential shocks, but the ratings decreased over time. This decrease is likely to be explained by increasing confidence that participants are able to avoid the shocks based on their performance in a given block (achieved by reaching RT higher faster than the mean RT from the training phase + 50 ms).

**Table S1.** Pupil dilation – full model results.

|  | Pupil dilation | |
| --- | --- | --- |
|  | **χ^2^**(df) | ***p*** |
| Threat condition | 34.30(2) | <.001 |
| Block (centered) | 18.60(1) | <.001 |
| Order (centered) | 12.50(1) | <.001 |
| Threat condition x Block (centered) | 27.91(1) | <.001 |

**Table S2*.*** Behavioral responses – full model results for reaction time (RT) and accuracy.

|  |  | RT | Accuracy | |
| --- | --- | --- | --- | --- |
|  | **χ^2^**(df) | ***p*** | **χ^2^**(df) | ***p*** |
| Threat condition | 26.93(2) | <.001 | 0.76(2) | .68 |
| Cue validity | 95.82(1) | <.001 | 2.40(1) | .12 |
| Block (centered) | .00(1) | .98 | 2.18(1) | .14 |
| Order (centered) | 53.74(1) | <.001 | 12.71(1) | <.001 |
| Threat condition x Cue validity | 1.10(2) | .58 | 0.44(2) | .80 |
| Threat condition x Block (centered) | 7.82(2) | .02 | 2.31(1) | .31 |
| Cue validity x Block (centered) | .09(1) | .76 | 7.05(1) | .008 |
| Threat condition x Cue validity x Block (centered) | 4.42(2) | .11 | 1.74(1) | .42 |

**Table S3.** Mean reaction time (RT) in each threat condition and for valid and invalid trials

|  | **Mean RT (SE)** | |
| --- | --- | --- |
| **Threat condition** | **Valid** | **Invalid** |
| Safe | 496.60 (11.04) | 520.47 (11.93) |
| Shock to Self | 475.97 (9.37) | 506 (9.54) |
| Shock to Other | 485.27 (10.17) | 510.51 (12.18) |

**Table S4*.*** Cue validity effect (RT and accuracy valid – invalid trials) – full model results

|  | RT | | Accuracy | |
| --- | --- | --- | --- | --- |
|  | **χ^2^**(df) | ***p*** | **χ^2^**(df) | ***p*** |
| Threat condition | 1.06(2) | .59 | 0.001 (2) | .99 |
| Block (centered) | 0.03(1) | .85 | 5.43 (1) | .02 |
| Order (centered) | 0.13(1) | .72 | 1.45 (1) | .23 |
| Threat condition x Block (centered) | 5.27(1) | .07 | 2.47(2) | .29 |
